# A terpenoids database with the chemical content as a novel agronomic trait

**DOI:** 10.1093/database/baae027

**Published:** 2024-05-22

**Authors:** Wenqian Li, Yinliang Chen, Ruofei Yang, Zilong Hu, Shaozhong Wei, Sheng Hu, Xinjun Xiong, Meijuan Wang, Ammar Lubeiny, Xiaohua Li, Minglei Feng, Shuang Dong, Xinlu Xie, Chao Nie, Jingyi Zhang, Yunhao Luo, Yichen Zhou, Ruodi Liu, Jinhai Pan, De-Xin Kong, Xuebo Hu

**Affiliations:** Institute for Medicinal Plants, College of Plant Science and Technology, Huazhong Agricultural University, Wuhan 430070, China; Innovation Academy of International Traditional Chinese Medicinal Materials, Huazhong Agricultural University, Wuhan 430070, China; National-Regional Joint Engineering Research Center in Hubei for Medicinal Plant Breeding and Cultivation, Huazhong Agricultural University, Wuhan 430070, China; Medicinal Plant Engineering Research Center of Hubei Province, Huazhong Agricultural University, Wuhan 430070, China; National Key Laboratory of Agricultural Microbiology, Agricultural Bioinformatics Key Laboratory of Hubei Province, College of Informatics, Huazhong Agricultural University, Wuhan 430070, China; National Key Laboratory of Agricultural Microbiology, Agricultural Bioinformatics Key Laboratory of Hubei Province, College of Informatics, Huazhong Agricultural University, Wuhan 430070, China; National Key Laboratory of Agricultural Microbiology, Agricultural Bioinformatics Key Laboratory of Hubei Province, College of Informatics, Huazhong Agricultural University, Wuhan 430070, China; Colorectal cancer clinical research center of HuBei Province，Colorectal cancer clinical research center of Wuhan, Hubei Cancer Hospital，Tongji Medical College, Huazhong University of Science and Technology，, Wuhan, Hubei 430069, China; Colorectal cancer clinical research center of HuBei Province，Colorectal cancer clinical research center of Wuhan, Hubei Cancer Hospital，Tongji Medical College, Huazhong University of Science and Technology，, Wuhan, Hubei 430069, China; Research Center for Rural Revitalization, Power China Kunming Engineering Corporation Limited, Kunming 650051, China; Shennongjia Academy of Forestry, Shennongjia, Hubei 442400 China; Africa City of Technology, Khartoum North, Sudan; Institute for Medicinal Plants, College of Plant Science and Technology, Huazhong Agricultural University, Wuhan 430070, China; Innovation Academy of International Traditional Chinese Medicinal Materials, Huazhong Agricultural University, Wuhan 430070, China; National-Regional Joint Engineering Research Center in Hubei for Medicinal Plant Breeding and Cultivation, Huazhong Agricultural University, Wuhan 430070, China; Medicinal Plant Engineering Research Center of Hubei Province, Huazhong Agricultural University, Wuhan 430070, China; Research Center for Rural Revitalization, Power China Kunming Engineering Corporation Limited, Kunming 650051, China; Institute for Medicinal Plants, College of Plant Science and Technology, Huazhong Agricultural University, Wuhan 430070, China; Innovation Academy of International Traditional Chinese Medicinal Materials, Huazhong Agricultural University, Wuhan 430070, China; National-Regional Joint Engineering Research Center in Hubei for Medicinal Plant Breeding and Cultivation, Huazhong Agricultural University, Wuhan 430070, China; Medicinal Plant Engineering Research Center of Hubei Province, Huazhong Agricultural University, Wuhan 430070, China; Institute for Medicinal Plants, College of Plant Science and Technology, Huazhong Agricultural University, Wuhan 430070, China; Innovation Academy of International Traditional Chinese Medicinal Materials, Huazhong Agricultural University, Wuhan 430070, China; National-Regional Joint Engineering Research Center in Hubei for Medicinal Plant Breeding and Cultivation, Huazhong Agricultural University, Wuhan 430070, China; Medicinal Plant Engineering Research Center of Hubei Province, Huazhong Agricultural University, Wuhan 430070, China; Institute for Medicinal Plants, College of Plant Science and Technology, Huazhong Agricultural University, Wuhan 430070, China; Innovation Academy of International Traditional Chinese Medicinal Materials, Huazhong Agricultural University, Wuhan 430070, China; National-Regional Joint Engineering Research Center in Hubei for Medicinal Plant Breeding and Cultivation, Huazhong Agricultural University, Wuhan 430070, China; Medicinal Plant Engineering Research Center of Hubei Province, Huazhong Agricultural University, Wuhan 430070, China; Institute for Medicinal Plants, College of Plant Science and Technology, Huazhong Agricultural University, Wuhan 430070, China; Innovation Academy of International Traditional Chinese Medicinal Materials, Huazhong Agricultural University, Wuhan 430070, China; National-Regional Joint Engineering Research Center in Hubei for Medicinal Plant Breeding and Cultivation, Huazhong Agricultural University, Wuhan 430070, China; Medicinal Plant Engineering Research Center of Hubei Province, Huazhong Agricultural University, Wuhan 430070, China; Institute for Medicinal Plants, College of Plant Science and Technology, Huazhong Agricultural University, Wuhan 430070, China; Innovation Academy of International Traditional Chinese Medicinal Materials, Huazhong Agricultural University, Wuhan 430070, China; National-Regional Joint Engineering Research Center in Hubei for Medicinal Plant Breeding and Cultivation, Huazhong Agricultural University, Wuhan 430070, China; Medicinal Plant Engineering Research Center of Hubei Province, Huazhong Agricultural University, Wuhan 430070, China; Institute for Medicinal Plants, College of Plant Science and Technology, Huazhong Agricultural University, Wuhan 430070, China; Innovation Academy of International Traditional Chinese Medicinal Materials, Huazhong Agricultural University, Wuhan 430070, China; National-Regional Joint Engineering Research Center in Hubei for Medicinal Plant Breeding and Cultivation, Huazhong Agricultural University, Wuhan 430070, China; Medicinal Plant Engineering Research Center of Hubei Province, Huazhong Agricultural University, Wuhan 430070, China; Institute for Medicinal Plants, College of Plant Science and Technology, Huazhong Agricultural University, Wuhan 430070, China; Innovation Academy of International Traditional Chinese Medicinal Materials, Huazhong Agricultural University, Wuhan 430070, China; National-Regional Joint Engineering Research Center in Hubei for Medicinal Plant Breeding and Cultivation, Huazhong Agricultural University, Wuhan 430070, China; Medicinal Plant Engineering Research Center of Hubei Province, Huazhong Agricultural University, Wuhan 430070, China; Institute for Medicinal Plants, College of Plant Science and Technology, Huazhong Agricultural University, Wuhan 430070, China; Innovation Academy of International Traditional Chinese Medicinal Materials, Huazhong Agricultural University, Wuhan 430070, China; National-Regional Joint Engineering Research Center in Hubei for Medicinal Plant Breeding and Cultivation, Huazhong Agricultural University, Wuhan 430070, China; Medicinal Plant Engineering Research Center of Hubei Province, Huazhong Agricultural University, Wuhan 430070, China; National Key Laboratory of Agricultural Microbiology, Agricultural Bioinformatics Key Laboratory of Hubei Province, College of Informatics, Huazhong Agricultural University, Wuhan 430070, China; Institute for Medicinal Plants, College of Plant Science and Technology, Huazhong Agricultural University, Wuhan 430070, China; Innovation Academy of International Traditional Chinese Medicinal Materials, Huazhong Agricultural University, Wuhan 430070, China; National-Regional Joint Engineering Research Center in Hubei for Medicinal Plant Breeding and Cultivation, Huazhong Agricultural University, Wuhan 430070, China; Medicinal Plant Engineering Research Center of Hubei Province, Huazhong Agricultural University, Wuhan 430070, China

## Abstract

Natural products play a pivotal role in drug discovery, and the richness of natural products, albeit significantly influenced by various environmental factors, is predominantly determined by intrinsic genetics of a series of enzymatic reactions and produced as secondary metabolites of organisms. Heretofore, few natural product-related databases take the chemical content into consideration as a prominent property. To gain unique insights into the quantitative diversity of natural products, we have developed the first TerPenoids database embedded with Content information (TPCN) with features such as compound browsing, structural search, scaffold analysis, similarity analysis and data download. This database can be accessed through a web-based computational toolkit available at http://www.tpcn.pro/. By conducting meticulous manual searches and analyzing over 10 000 reference papers, the TPCN database has successfully integrated 6383 terpenoids obtained from 1254 distinct plant species. The database encompasses exhaustive details including isolation parts, comprehensive molecule structures, chemical abstracts service registry number (CAS number) and 7508 content descriptions. The TPCN database accentuates both the qualitative and quantitative dimensions as invaluable phenotypic characteristics of natural products that have undergone genetic evolution. By acting as an indispensable criterion, the TPCN database facilitates the discovery of drug alternatives with high content and the selection of high-yield medicinal plant species or phylogenetic alternatives, thereby fostering sustainable, cost-effective and environmentally friendly drug discovery in pharmaceutical farming.

**Database URL**: http://www.tpcn.pro/

## Introduction

Natural products play a vital role as a source of innovative drugs according to numerous scientific studies (1–3). Terpenoids are the most abundant class of natural products, from hemiterpenes and monoterpenes with very low molecular weight (MW) to triterpenes and tetraterpenes with largeMWs, exhibiting linear, planar molecules to complex three-dimensional bridge and ring structures (4, 5). Compared with other natural products such as flavonoids and phenylpropanoids, the structure, quantity and activity of terpenes are more diverse (5, 6). Terpenoids play an important role in drug discovery and pharmaceutical fields due to their enormous structural and physicochemical diversities.

Nearly 76% of terpenoids are derived from plants (7), and they are one of the main groups of bioactive compounds in medicinal plants (6). Potent and active terpenoids such as andrographolide (bacillary dysentery) (8), paclitaxel (anticancerous) (9), artemisinin (antimalarial) (10), triptolide (anti-inflammatory) (11), asiaticoside (vulnerary) (12) and paeoniflorin (anti-inflammatory) (13) are derived from plants. Presently, ∼70 000 different plants are used by traditional and modern medical systems worldwide (14). In China, around 329 species of medicinal herbs are cultivated on >5.56 million hectares (15). According to the World Health Organization, the current global market value for medicinal plants stands at $14 billion per annum and will exceed to $5 trillion by 2050 (16). These estimations highlight the substantial growth projected in the demand for medicinal plants over the coming decades.

The content of natural products is affected by various factors. Recently, research found that the content of the same natural product can vary under different conditions, such as molecular regulation, species factor, environmental condition and combined factors (17–19). In the evolutionary formation of natural products, there are characteristics of convergent evolution (20). Unrelated plants may evolve the same natural products or different compounds with functionally similar properties, due to environmental stress involving genetic changes (21–23). Taking advantage of the considerable difference in the abundances of structurally similar compounds, a low-content natural product can be substituted with another compound that shares similar functions but can be obtained in higher yields from the original plant (24).

In order to align with the accelerated pace of contemporary drug discovery and meet the growing demand for pharmaceutical raw materials, experts in natural product science must consistently enhance both the quality and quantity of active compounds (25). The composition and concentration values of natural products facilitate the selection of active compounds of high content, and they determine the quality of herbal medicines, which would help to select high-yield germplasm resources (26). Several terpenoid databases have been reported with chemical structures, biological sources, bioactivity and terpene synthases, but few databases described the data on terpenoids content variations specifically (7, 27, 28). In this study, we summarize the species, content and tissue origin of terpenes isolated from plants between 1946 and 2022, including many active compounds, and establish a content-embedded database of terpenoids (TPCN), which is accessible through a web-based computational toolkit available at http://www.tpcn.pro/. The TPCN included the yield of secondary metabolites, the key target phenotypic trait of medicinal plants, as an important reference basis to facilitate the discovery of drug alternatives with enhanced content for higher druggability and assist in the screening of high-quality medicinal plant lines or identifying new alternative lines.

## Materials and methods

### Data sources

**Figure 1.**
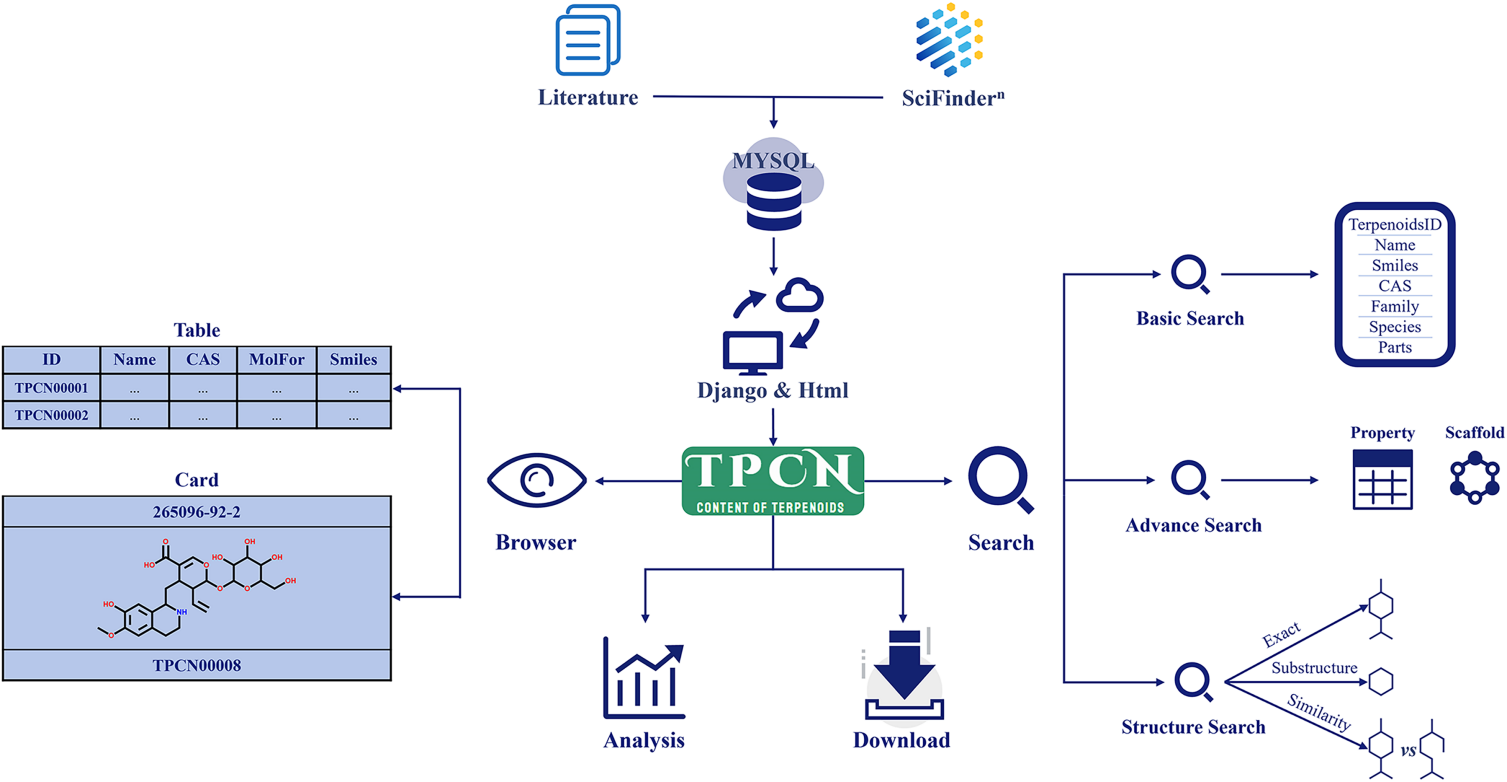
The architecture of TPCN.

All data in the TPCN were extracted from the literature and various online database resources (Figure 1). The Web of Science was searched using keyword combinations like ‘terpenoids’, ‘monoterpenes’, ‘diterpenes’, ‘sesquiterpenes’ and ‘triterpenes’ to collect literature on the content of terpenoids from 1946 to 2022. Then, the content information of terpenoids was recorded and input into the database manually, including chemical names, biological sources (family and species), extraction parts, content values and literature sources. The structure of terpenoids was extracted from SciFinder and standardized using RDKit (29). To ensure the accuracy of the structures, we compared the structures from literature and SciFinder, recording solely the matching structural information.

### Data distribution

Relying on manual collection and sorting of literature data, the distribution of terpenoids from different perspectives was analyzed, including structural type, biological source, extraction part and content. These terpenoids consisted of four categories: monoterpenoids, sesquiterpenoids, diterpenoids and triterpenoids, with the respective counts calculated for each category. A more comprehensive analysis of the distribution of terpenoids from various biological sources (family and species) and extraction parts was also conducted. In addition, to conduct a thorough analysis of the content distribution of terpenoids, we segmented the content into seven ranges: 10^–6^% to 10^–5^%, 10^–5^% to 10^–4^%, 10^–4^% to 10^–3^%, 10^–3^% to 10^–2^%, 10^–2^% to 10^–1^%, 10^–1^% to 1% and 1% to 10%. The number of terpenoids in each content range was counted. It is noteworthy that the classification information of terpenoids was initially extracted from the literature and then incorporated into the database. When extracting 1 g of terpenoids from 1 kg of raw material, the content is expressed as 0.1% (i.e. 10^–1^%).

### Similarity calculation

Similarity measure comprises three essential components: molecular representation, weighting scheme and similarity coefficient (30). The Tanimoto coefficient is extensively utilized in chemoinformatics and computational medicinal chemistry owing to its computational simplicity and rapid processing speed. Nonetheless, it also demonstrates a certain level of reliance on the sizes of the molecules, resulting in reduced similarity values particularly when searching for small reference structures (where only a few bits are activated in the reference structure’s fingerprint) (31). The Dice coefficient is also extensively utilized to measure molecular similarity due to its simplicity in calculations, yet it is comparatively slower computationally compared to the Tanimoto coefficient (32). The Cosine coefficient is frequently employed to gauge similarity between sparse data and can rapidly calculate the average similarity between all pairs of compounds in the datasets (33). Within the similarity search interface of TPCN, four molecular fingerprints (Daylight fingerprint, ECFP4, ECFP6 and MACCS) and three similarity indices (Tanimoto/Jaccard coefficient, Dice coefficient and Cosine coefficient) could be selected to calculate the similarity between molecules using RDKit. Unless otherwise specified, daylight fingerprint and Tanimoto coefficient were applied to calculate the similarity between terpenoids. Additionally, the content differences of terpenoids with structural similarity over 0.95 were also further calculated. Notably, in the case of terpenoids with multiple content values, the content difference was calculated by the maximum content value for each terpenoid.

### Murcko scaffold analysis

Murcko scaffold is the core structure of a molecule that is composed of the ring systems and the linkers between them. Double bonds directly attached to the ring systems, or linkers are retained (Supplementary Figure S1) (34). To further explore the relationship between terpenoid scaffolds and their content, the dominant Murcko scaffolds (ordered by the occurrence frequency) of terpenoids from different content ranges were generated by RDKit. Initially, we categorized terpenoids into seven groups based on their content ranges. Subsequently, we generated the Murcko scaffolds for each group of terpenoids and recorded the occurrence frequency of each scaffold. Lastly, the top 10 dominant Murcko scaffolds for each content range were displayed. Besides, to explore the relationship between the content of terpenoids and their glycosylation levels, the glycosylation ratio of terpenoids from different content ranges was counted. Sugar Removal Utility (SRU), a tool for deglycosylation, was used for the identification and removal of sugar moieties of terpenoids (35). The parameters of the SRU were set as follows. The circular and linear sugar moieties, as well as non-terminal and terminal sugar moieties, were all removed. The fragments with fewer than five heavy atoms that got disconnected from the molecule after the removal of sugar moieties were removed. The minimum ratio of circular sugar between its exocyclic oxygen atoms and the atoms within the sugar ring was set to 0.4. Other parameters were set as the default values (36).

### Physicochemical property calculation

To explore the differences in the physicochemical properties of terpenoids from different content ranges, we calculated 11 physicochemical properties of terpenoids by RDKit. These physicochemical properties are MW, hydrogen bond acceptor (HBA), hydrogen bond donor (HBD), octanol–water partition coefficient (AlogP), topological polar surface area (TPSA), number of rotatable bonds (NumRotatableBonds) (conjugated single bonds were not considered), number of heavy atoms (NumHeavyAtoms), number of aromatic rings (NumAromaticRings), number of aliphatic rings (NumAliphaticRings), number of rings (NumRings) and fraction of Csp3 atoms (FractionCsp3). The average value of the physicochemical properties of terpenoids from different content ranges was calculated.

## Results and discussion

### Analysis of the content of terpenoids with species and tissue sources

**Figure 2.**
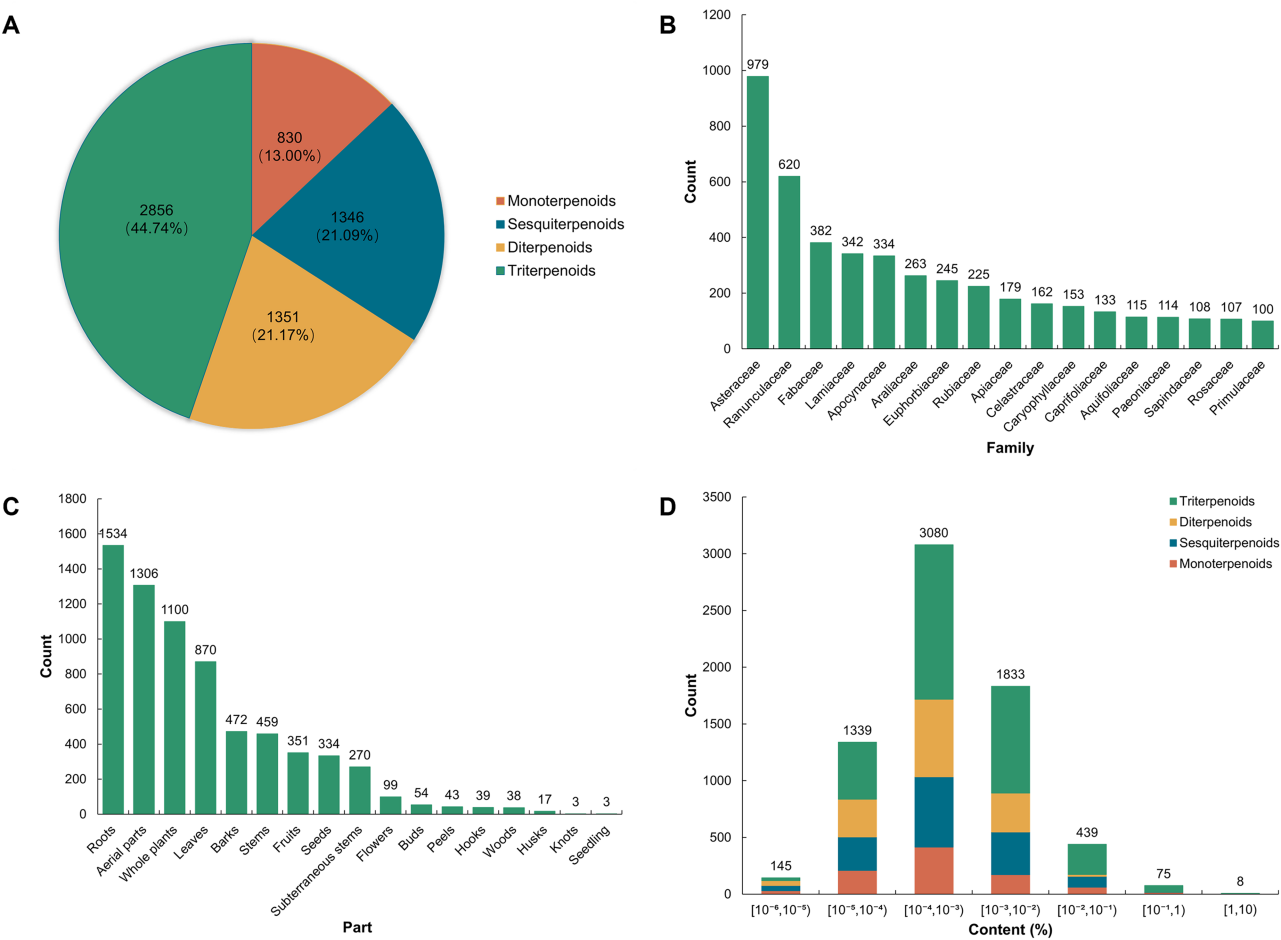
Distribution of terpenoids in the TPCN database based on (A) structural type, (B) family, (C) extraction part and (D) content range.

TPCN is the first content-embedded database of terpenoids, comprising 7508 content records for 6383 unique terpenoids. Based on the structural characteristics of terpenoids, the terpenoids in the TPCN database were classified into four categories: monoterpenoids, sesquiterpenoids, diterpenoids and triterpenoids. Triterpenoids (2856, 44.74%) are the predominant category of terpenoids in the TPCN database, followed by diterpenoids (1351, 21.17%), sesquiterpenoids (1346, 21.09%) and monoterpenoids (830, 13.00%) (Figure 2A).

**Figure 3.**
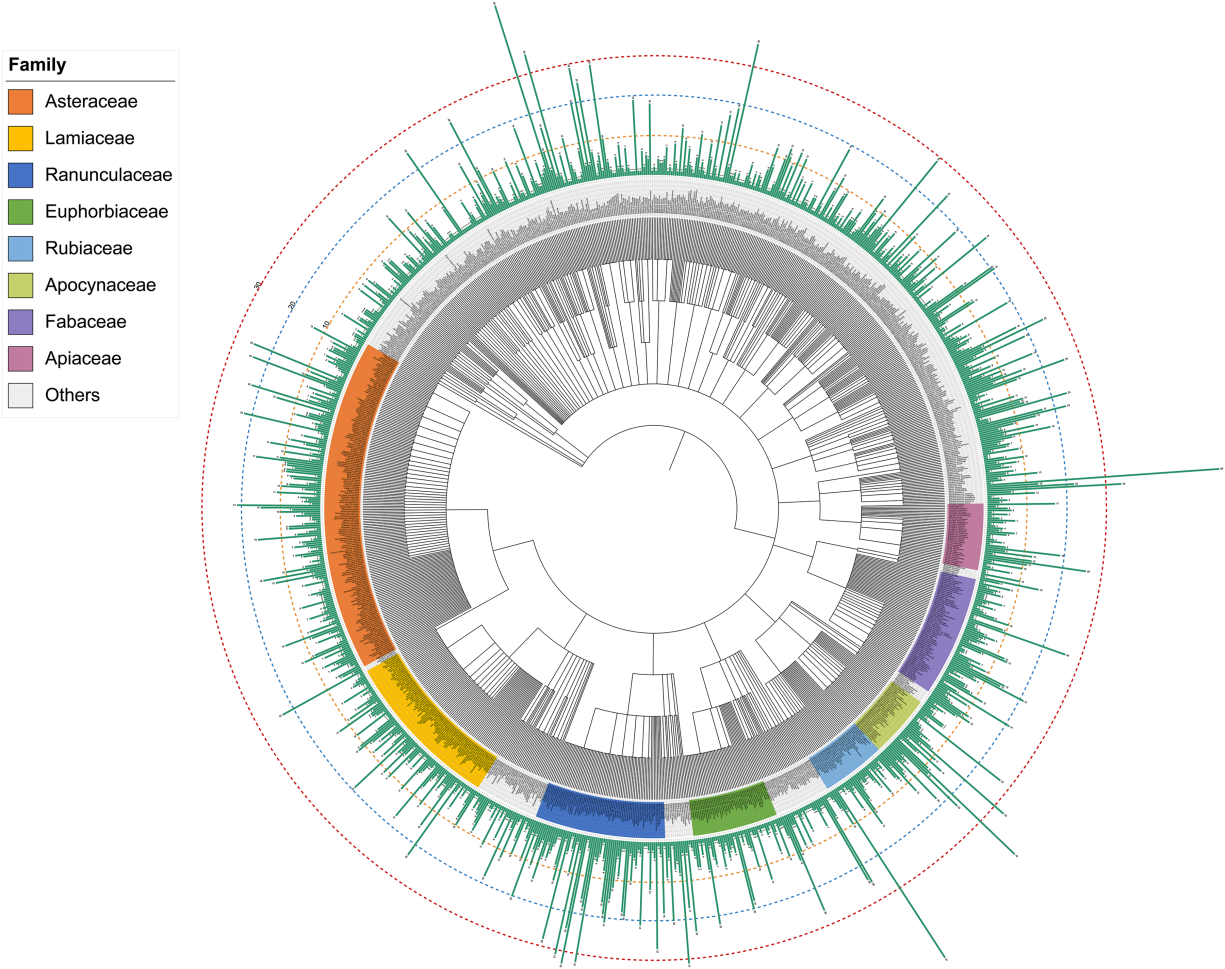
Schematic of the distribution of terpenoids across plant phylogeny.

Plants are an extraordinary source of bioactive molecules, and ∼76% of terpenoids are derived from plants (7). In the TPCN database, these terpenoids were derived from 1254 species belonging to 156 different plant families (Figure 3). *Asteraceae* (979, 15.34%) was the main source of terpenoids, followed by *Ranunculaceae* (620, 9.71%), *Fabaceae* (382, 5.98%) and *Lamiaceae* (342, 5.36%) (Figure 2B). Consistent with previous studies, terpenoids are one of the most important components of constituents among the secondary metabolites identified from *Asteraceae* (37).

Many terpenoids show specificity to certain species and tissues, indicating species-specific and tissues-specific functions (38–41). In addition, we also divided the extraction parts of these terpenoids into 17 types. According to the statistical results, the number of terpenoids from the root (1534, 24.03%) was the largest, followed by aerial parts (1306, 20.46%), whole plants (1100, 17.23%) and leaves (870, 13.63%). However, terpenoids derived from seedlings (3, 0.05%) and knots (3, 0.05%) were infrequently found (Figure 2C). In general, the root is the most predominant accumulation organ of terpenoids.

The production of natural products is important for functional research and commercial development (42). But, for the vast majority of natural products, it is the most important factor which limits their further development and research due to their low content in plant tissues and the long growth cycle of plants (43). In the TPCN database, the content of terpenoids ranged from 0.000001% to 3.744898%. We divided these terpenoids into seven distinct content ranges. The majority of terpenoids were predominantly present within the content range of 10^–4^% to 10^–3^% (Figure 2D), which is about a few parts per million.

**Figure 4.**
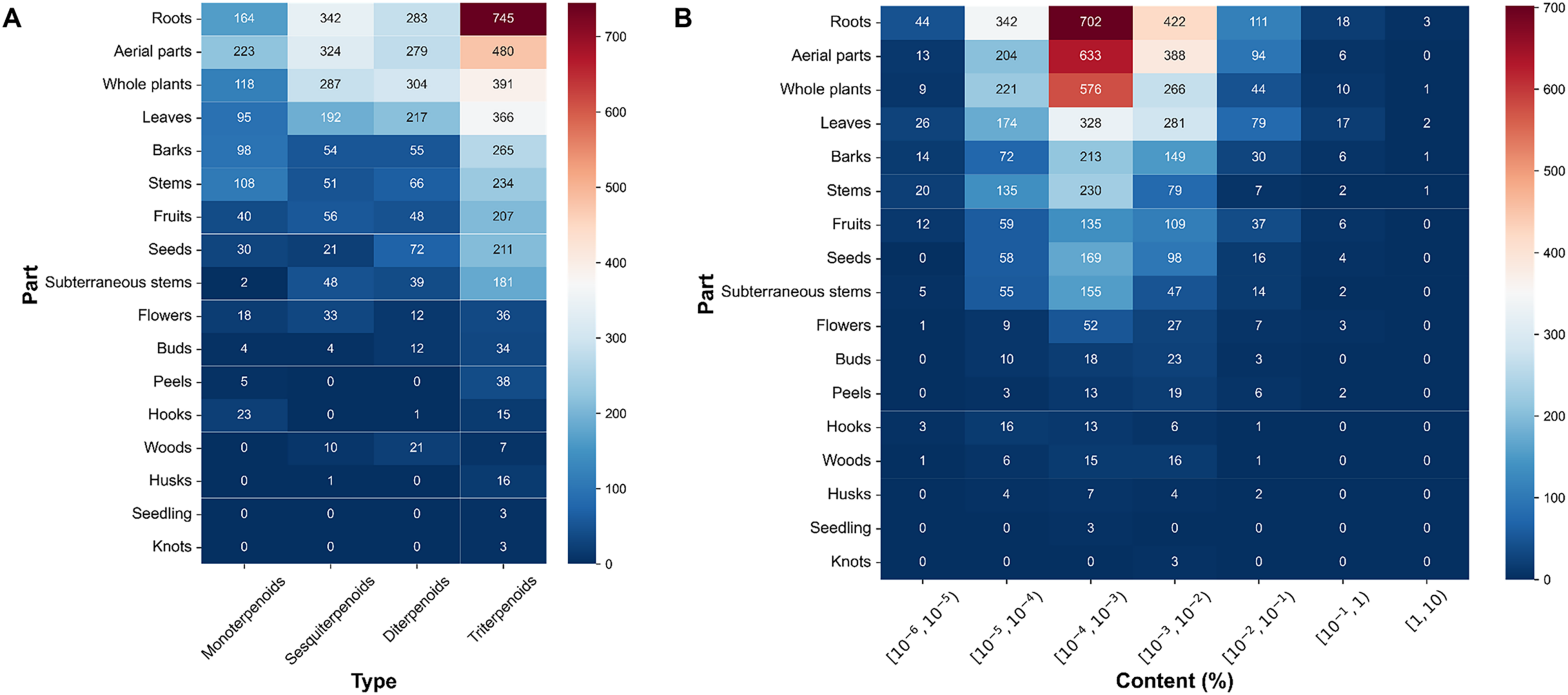
The plant tissues and content distribution of terpenoids. (A) The plant tissues distribution of various terpenoids. (B) The content distribution of terpenoids in each part.

Subsequently, we conducted further investigations into the number and content distribution of terpenoids in different extraction parts of plants. The results indicated that monoterpenoids were mainly derived from the aerial parts, while sesquiterpenoids exhibited significant distribution in both roots and aerial parts. Diterpenoids primarily originated from whole plants, and triterpenoids were prominently distributed in the roots of plants (Figure 4A). Regarding the content of different extraction parts of plants, the content of the majority of terpenoids was between 10^–5^% and 10^–2^%. Particularly, in the case of terpenoids derived from roots and aerial parts of plants, the content of terpenoids in these parts was mostly between 10^–4^% and 10^–3^% (Figure 4B).

### The structural similarity calculation and application of higher-content compounds

Most of the high-value natural products have usually low natural abundance and tedious chemical synthesis, which hinder their clinical translation. Structural similarity is one of the key strategies for drug discovery (44). Higher-content compounds that are structurally similar to other high-value compounds have the modification potential to wider applications (24).

**Figure 5.**
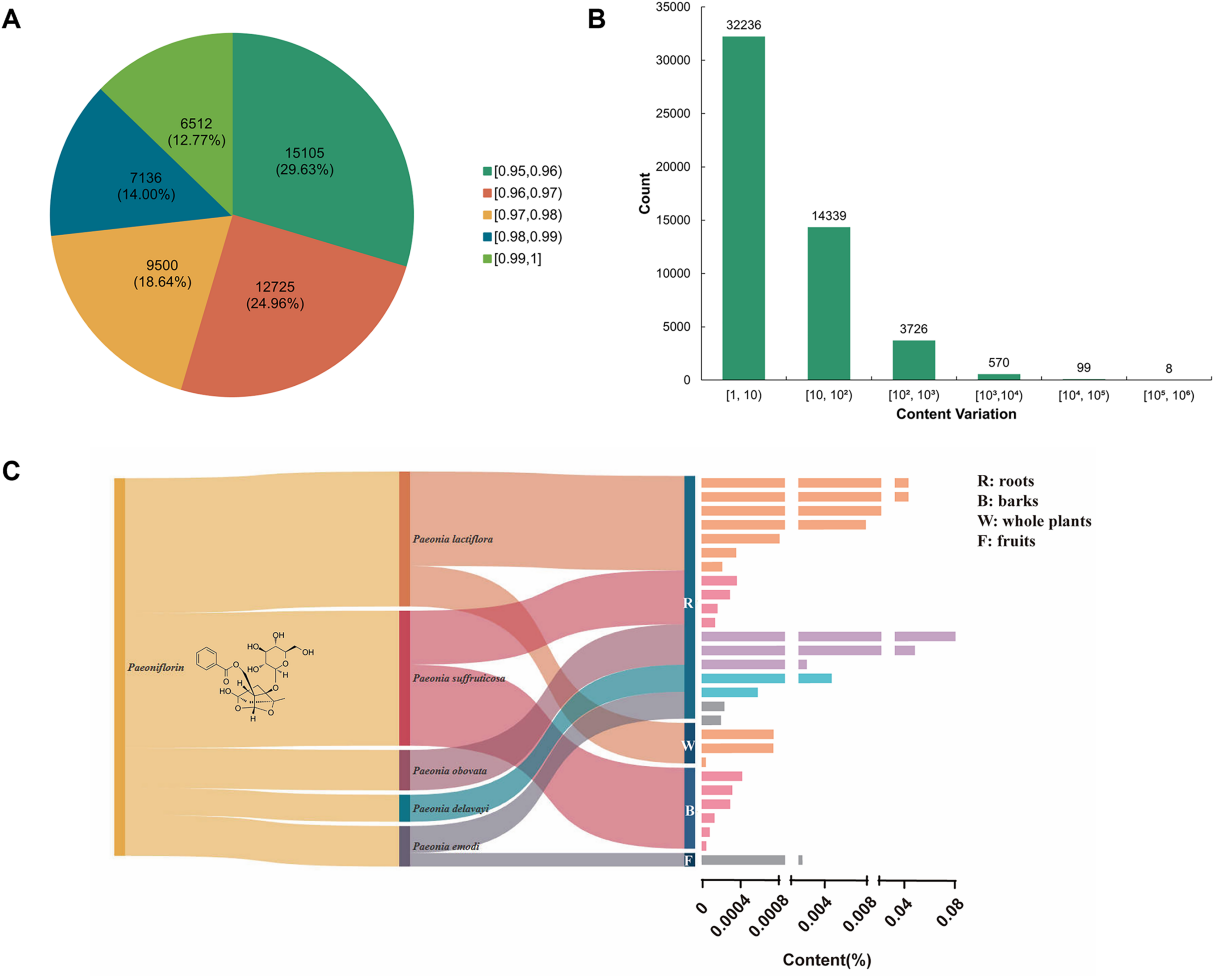
The distribution and content variation of structurally similar terpenoids. (A) The distribution of terpenoid compound pairs with structurally similar exceeding 0.95. (B) Content ratio between terpenoids with structurally similar exceeding 0.95. (C) The host source, extraction parts and content distribution of terpenoids with structural similarity exceeding 0.95 compared with paeoniflorin.

To analyze the structural similarity of terpenoids, we utilized RDKit to generate the daylight fingerprint of terpenoids and determine the correlation between them using the Tanimoto coefficient. Our results revealed that out of the examined pairs of terpenoids, 50 978 had a structural similarity of >95% and 6512 pairs exhibited a structural similarity exceeding 99% (Figure 5A).

Additionally, we measured the content variation among structurally similar terpenoids. The majority of these compounds showed a content variation of less than a 10-fold magnitude. However, there were eight pairs of terpenoids that displayed a structural similarity exceeding 95%, while exhibiting content variations surpassing magnitudes of 100 000 (Figure 5B). For example, despite glycyrrhizin and uralsaponin R sharing a structural similarity of 0.9728, their content variation reached an astonishing magnitude of 166 667 (Table 1).

**Table 1.** The variation of terpenoid content with structural similarity exceeding 0.95

CAS number^a^	Structure^a^	Species^a^	Part^a^	Content (%)^a^	CAS number^b^	Structure^b^	Species^b^	Part^b^	Content (%)^b^	Similarity	Fold^ab^
1405-86-3	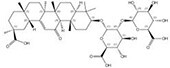	*Glycyrrhiza inflata*	Roots	1.500000	1616062-84-0	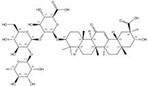	*Glycyrrhiza uralensis*	Roots	0.000009	0.9728	166 667
57817-89-7	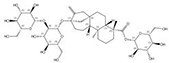	*Stevia rebaudiana*	Leaves	2.522321	64849-39-4	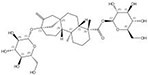	*Orychophragmus violaceus*	Seeds	0.000018	0.9674	140 129
1405-86-3	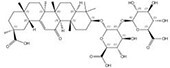	*Glycyrrhiza inflata*	Roots	1.500000	1616062-82-8	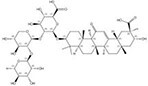	*Glycyrrhiza uralensis*	Roots	0.000011	0.9728	136 364
1405-86-3	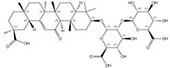	*Glycyrrhiza inflata*	Roots	1.500000	1616062-79-3	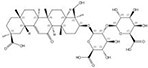	*Glycyrrhiza uralensis*	Roots	0.000011	0.9828	136 364
1422049-84-0	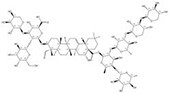	*Acanthophyllum gypsophiloides*	Roots	3.744898	1704595-03-8	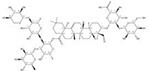	*Momordica charantia*	Seeds	0.000033	0.9680	113 482
121340-61-2	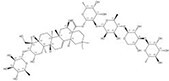	*Bellis sylvestris*	Whole plants	2.600000	1028100-30-2	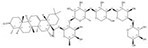	*Gypsophila oldhamiana*	Roots	0.000023	0.9544	113 043
1405-86-3	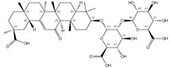	*Glycyrrhiza inflata*	Roots	1.500000	1616062-88-4	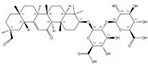	*Glycyrrhiza uralensis*	Roots	0.000014	0.9790	107 143
1405-86-3	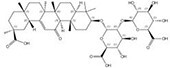	*Glycyrrhiza inflata*	Roots	1.500000	1616062-83-9	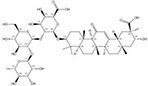	*Glycyrrhiza uralensis*	Roots	0.000014	0.9728	107 143

Superscript a and b represent two different terpenoids (compound a and compound b), respectively. Fold^ab^ represents the content ratio of compound a and compound b. This table shows the compound pairs with a structural similarity of 0.95 or higher and the content ratio >10 000 in the TPCN.

Notably, we observed that even closely related species can produce similar chemicals with significant differences in content. As an illustration, *Glycyrrhiza inflata* contains a higher content of glycyrrhizin compared to the genetically proximate species *G. uralensis*, which possesses five structurally similar counterparts but with extremely low abundance (Table 1). Similarly, *Bellis sylvestris* (nomenclature family: *Asteraceae*, order: *Asterales*, class: *Magnoliopsida*) and *Gypsophila oldhamiana* (nomenclature family: *Caryophyllaceae*, order: *Caryophyllales*, class *Magnoliopsida*), despite having a distant taxonomic relationship, both produce very similar chemicals (Table 1). Using information as such, we were able to identify alternatives to celastrol that exhibit improved drug-like properties, along with increased availability and reduced toxicity (24).

Paeoniflorin is mainly extracted from the Paeoniaceae plants (45, 46). Modern medical studies have shown that paeoniflorin has immunoregulatory, antidepressant, anti-arthritis, antithrombosis, anti-tumor, hepatoprotective, cerebral ischemic injury protective and neuroprotective effects (13, 47). However, paeoniflorin is of low yield and has difficulty in separation of extracts. Moreover, the biosynthesis pathway of paeoniflorin has not been fully elucidated (45), which fundamentally limits the production of paeoniflorin by synthetic biology. Compounds with similarity between 0.95 and 1 compared with paeoniflorin were distributed among five species of the genus *Paeonia*, and the higher-content compounds could be modified to obtain similar functions to paeoniflorin with higher abundance (Figure 5C).

Similar to the complexity of crop yields (48), the content of a natural product is essentially the result of a cascade of enzymatic catalyzing reactions, which are genetically encoded. However, it should be noted that environmental factors, such as temperature, light and soil composition, as well as human actions like the timing of harvest, processing and storage, also exert a significant impact on the content. We have retained all the original content information in our database. For instance, paeoniflorin’s content in the roots of *Paeonia lactiflora* from different producing areas exhibited variations of over 300 times from four independent investigations. The content of paeoniflorin in two materials from China closely resembled each other but was significantly lower than the measurements of the other two materials from Vietnam, which also exhibited similar content either (Supplementary Figure S2). In another example, oleanolic acid (OA), a triterpenoid, exists in numerous plant species with content differences of ∼2-fold within the same organs in *Eriope blanchetii* (Supplementary Figure S3). Furthermore, the major pharmaceutical components showed greater accumulation in *Artemisia annua* after graphene treatment, suggesting that the graphene-based cultivation strategy offers a novel solution to the problem of low artemisinin content, and the graphene could serve as a nanofertilizer to replace chemical fertilizer and decrease non-point-source pollution derived from agriculture. It is a promising strategy for the cultivation of medicinal plants environmentally friendly. Thus, the high level of compounds may inspire us to select more efficient and environmentally sustainable cultivation methods (49). Clearly, these significant variations may pique the interest of researchers working on these plants, prompting further investigations into the underlying biological or abiotic factors contributing to such differences.

### Scaffold and physicochemical properties of higher-content compounds

**Figure 6.**
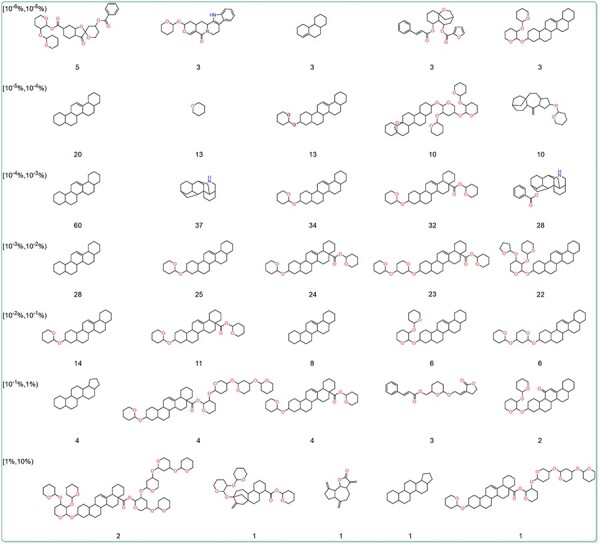
The dominant Murcko scaffold of terpenoids in different content ranges. The numbers represent the count of terpenoids with the scaffold.

To further explore the relationship between the content and structure of terpenoids, the Murcko scaffolds of terpenoids in different content ranges were generated using RDKit. The top five Murcko scaffolds with the highest frequency in various content ranges were shown (Figure 6). The results showed that 1,2,3,4,4a,5,6,6a,6b,7,8,8a,9 101 112,12a,12b,13,14b-icosahydropicene was the most frequently occurring scaffold structure among terpenoids across diverse content ranges. This is related to the fact that triterpenoids make up the majority of terpenoids in the database. Additionally, terpenoids with higher content tended to exhibit more oxygenated furan rings and oxygenated pyran rings. These structures are the core of many sugars and sugar-like units. To further investigate the content and glycosylation ratio of terpenoids, the glycosylation ratio of terpenoids from different content ranges was calculated. The findings revealed a positive correlation between the content of terpenoids and their glycosylation levels (Table 2). This could be attributed to the fact that glycosylation can enhance the water solubility and stability of terpenoids, thereby facilitating their storage.

**Table 2.** The glycosylation ratio of terpenoids in different content ranges

Content (%)	All	Glycosides	Percentages
[10^–6^,10^–5^)	145	56	38.62
[10^–5^,10^–4^)	1339	534	39.88
[10^–4^,10^–3^)	3080	1397	45.36
[10^–3^,10^–2^)	1833	981	53.52
[10^–2^,10^–1^)	439	272	61.96
[10^–1^,1)	75	62	82.67
[1,10)	8	5	62.50

**Figure 7.**
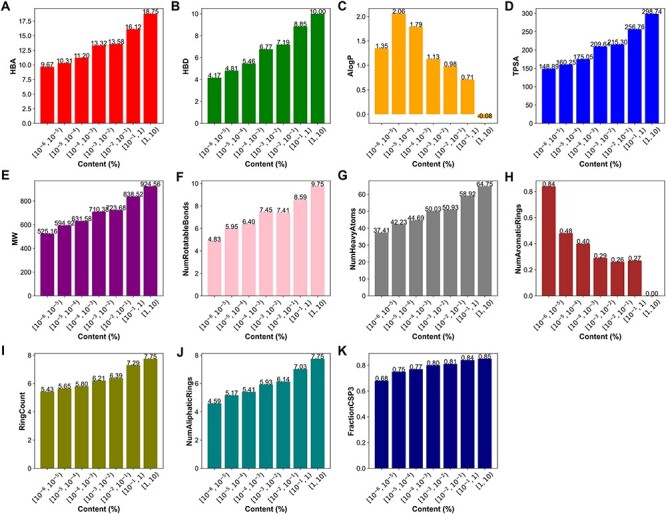
Physicochemical properties of terpenoids in different content ranges. (A) The hydrogen bond acceptors, HBA. (B) The hydrogen bond donors, HBD. (C) The octanol-water partition coefficient, AlogP. (D) The topological polar surface area, TPSA. (E) The molecular weight, MW. (F)The number of rotatable bonds, NumRotatableBonds. (G) The number of heavy atoms, NumHeavyAtoms. (H) The number of aromatic rings, NumAromaticRings. (I) The Ring Count, RingCount. (J) The number of aliphatic rings, NumAliphaticRings. (K) The fraction of sp3 hybridized carbons, FractionCSP3.

We also conducted an in-depth analysis of the relationship between the content and physicochemical properties of terpenoids. The 11 physicochemical properties of terpenoids were determined using RDKit. The results demonstrate that certain physicochemical properties correlated with molecular size and complexity, including HBA (Figure 7A), HBD (Figure 7B), TPSA, MW, NumRotatableBonds, NumHeavyAtoms (Figure 7D–G), FractionCsp3 (Figure 7K) as well as the RingCount (Figure 7I), and NumAliphaticRings (Figure 7J), showed a positive correlation with the content of terpenoids. However, the NumAromaticRings (Figure 7H) and AlogP (Figure 7C) negatively correlated with the content of terpenoids. This indicates that the larger, more complex, and more hydrophilic a compound is, the higher its content may be. It may be attributed to the introduction of sugar units through glycosylation modification in terpenoids.

### Examples of TPCN with ginsenosides

**Figure 8.**
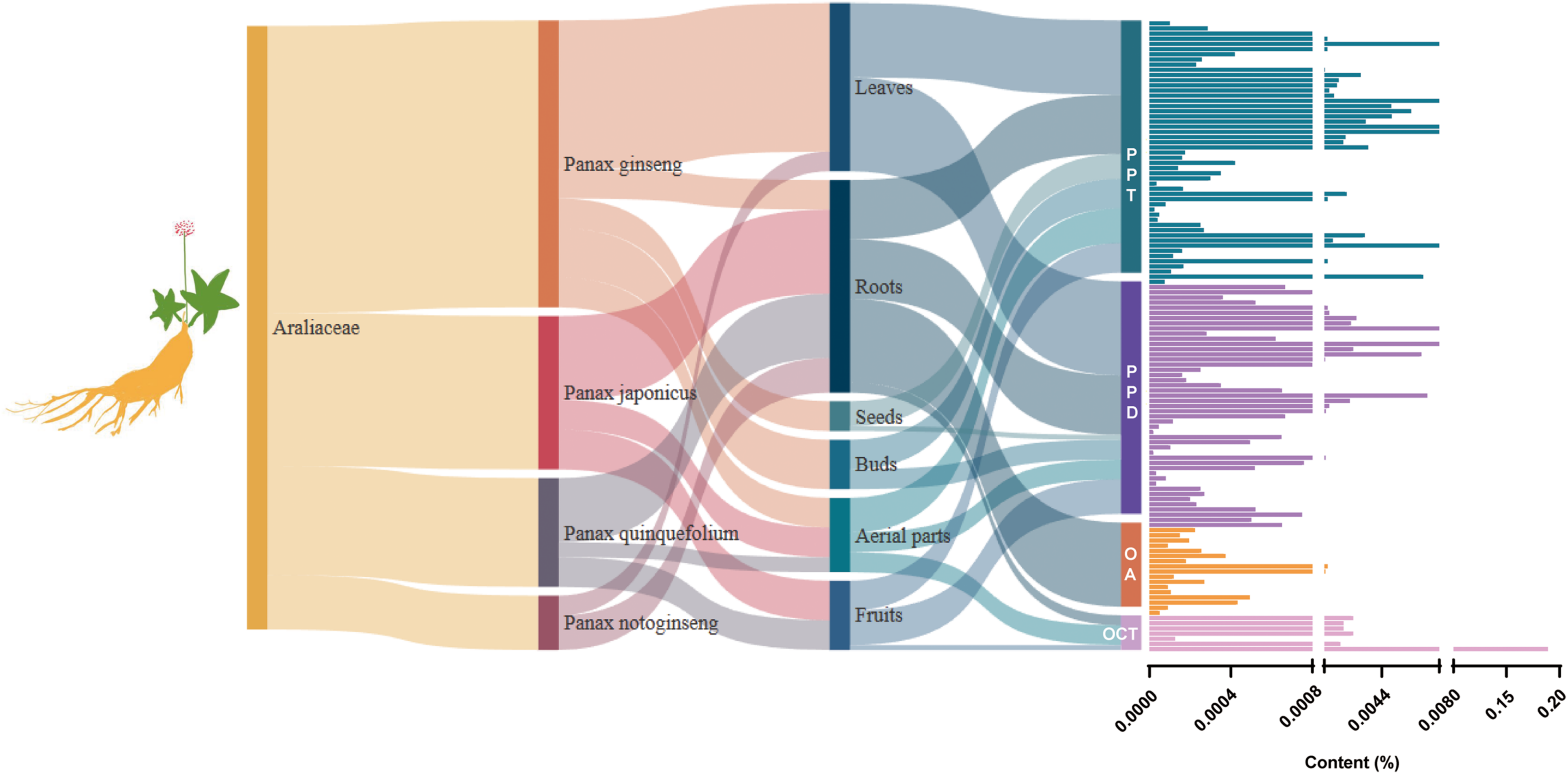
The host source, extraction parts and content distribution of ginsenosides.

Ginsenosides are specialized triterpene saponins uniquely present in the *Panax* species (48). Among the species of *P*. genus (50), *P. ginseng* (51), *P. notoginseng* (52), *P. quinquefolius* (53) and *P. japonicus* (54) have been widely used as medicinal and functional food. At present, most of the isolated ginsenosides can usually be divided into Dammarane type (DM type), OA type and Ocotillol type (OCT type) according to the structural differences of their glycosides. According to the difference in the hydroxyl ligand at the C6 position, DM-type ginsenosides are divided into protopanaxadiol-type (PPD-type) ginsenosides and protopanaxatriol-type (PPT-type) ginsenosides (55). Among all ginsenosides, tetracyclic triterpene DM saponins accounted for the majority of ginsenosides. Among the saponins isolated in ginseng, PPD-type ginsenosides have the most types and the highest content, followed by PPT-type, and OA-type ginsenosides have the least types and lowest content. The higher content of ginsenosides Rb1, Rb2, Rc, Rd, Re and Rg1 (the main ginsenosides, accounting for >80% of the total ginseng saponins) contains more saccharide groups and are more hydrophilic, but their biological activity is low, and the absorption rate in the human body is also very low (Figure 8). Rare ginsenosides (Rh2, Rg3, etc.) contain less glycosyl, have better biological activity and higher body absorption rate and play a significant role in regulating metabolism, promoting cell differentiation and resisting tumors (56, 57); however, their content in natural ginseng plants is very low. The types and contents of ginsenosides contained in different species of ginseng plants are different, and the content of ginsenosides in the same ginseng plants is also very different. The main active ingredients of *P. ginseng, P. quinquefolius, P. notoginseng* and other medicinal materials are DM-type saponins, while the main active ingredients of *P. japonicus* are OA-type ginsenosides and contain a small amount of DM-type ginsenosides (Figure 8).

Thus, different genotypes of ginseng plants influence the ginsenoside type and content. Although *P. quinquefolius, P. ginseng* and *P. notoginseng* are morphologically and phylogenetically close, each ginseng species contains characteristic types and/or levels of ginsenosides. These differences among various ginseng species reflect the genetic diversity in synthesis and accumulation of ginsenosides in different ginseng species.

### Web interface of TPCN

**Figure 9.**
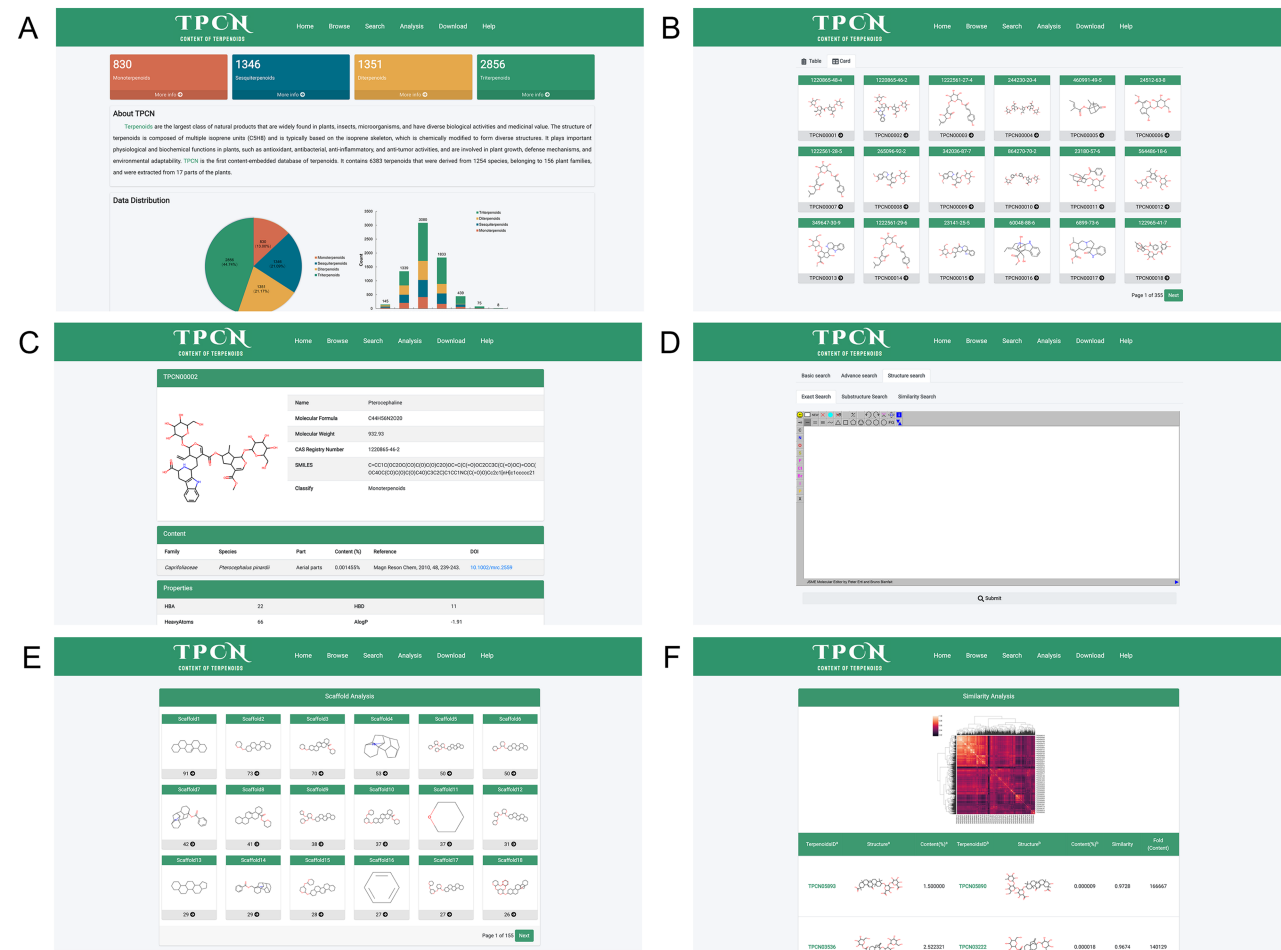
The web pages of TPCN database. (A) Home; (B) Browse; (C) Compound detail; (D) Structural search; (E) Scaffold analysis; (F) Similarity analysis.

In order to facilitate the application of this database and to continually expand the amount of data and add more information, we have hosted this database on the website (http://www.tpcn.pro/). TPCN was designed to include home, browse, search, analysis, download and help document interfaces. The home interface provides an overview of the introduction, data composition and data sources of the database. In addition, it also enables users to browse a specific category of terpenoids by clicking on the corresponding module (Figure 9A). The browse interface consists of table browse and card browse (Figure 9B). Users can browse the detailed information of that compound by clicking on the respective molecular image, including its structure, content and physicochemical properties (Figure 9C). The search interface allows users to utilize various search criteria to retrieve relevant compounds, including basic information, physicochemical properties, Murcko scaffold and the structure of terpenoids. The basic information search encompasses several key components, namely, the name, smiles, chemical abstracts service registry number (CAS number), molecular formula, biological source (family and species), extraction part and classification of terpenoids. The physicochemical properties search and Murcko scaffold search provide users with the ability to narrow down their search for target compounds based on specific physicochemical properties or Murcko scaffold. The structure search involves three distinct search modes: exact search, substructure search and similarity search. A plugin (58) for chemical structure drawing is integrated into the web page, which can be used for structural searching (Figure 9D). The analysis interface displays all the Murcko scaffolds of terpenoids in the database (Figure 9E), as well as the similarity and content variations of terpenoids with structural similarity exceeding 0.95 (Figure 9F). The download interface allows users to download the structures of the terpenoids as well as their species sources. The detailed functionality and usage of the database are provided in the help document interface.

## Conclusion

Terpenoid natural products exhibit intricate molecular structures and possess immense potential for pharmacological applications, making them a highly valuable resource for drug discovery. Despite the extensively documented efficacy of plants as sources of terpenoids, the sustainable and economically feasible production of most of these compounds in significant quantities remains a formidable challenge, particularly in cases where extraction from plants is necessary. Consequently, attaining high yields of natural products becomes a pivotal factor in augmenting agricultural productivity and fostering environmental sustainability. In response to this challenge, the comprehensive platform TPCN has been devised. TPCN presently serves as the most extensive repository of comprehensive data on molecule structures, biological sources and extraction methods, offering significant assistance to researchers in the meticulous selection of suitable species for breeding, extraction of phytochemicals and identification of alternative candidates for drug discovery derived from natural products. The development of TPCN shall also advance our understanding of the fundamental biosynthetic mechanisms underlying natural products and, more specifically, their chemical diversity, encompassing both qualitative and quantitative aspects as invaluable phenotypic characteristics that have progressively evolved over time. For economically significant natural product–based drugs and their alternatives, the heightened chemical content found in plants represents a heritable trait stemming from the efficiency of photosynthesis and secondary metabolic transformations. This attribute confers substantial benefits in terms of eco-friendliness, cost-effectiveness and practicality within the realm of pharmaceutical agriculture.

## Supplementary Material

baae027_Supp

## Data Availability

All the data used in this manuscript are collected from the literature and various online database resources and can be freely accessed or downloaded by visiting our website (http://www.tpcn.pro/). The source code used in this analysis is available from https://github.com/ylchen0622/TPCN.
