# Correction to: A Terpenoids Database with the Chemical Content as A Novel Agronomic Trait

**DOI:** 10.1093/database/baae050

**Published:** 2024-06-19

This is a correction to: Wenqian Li, Yinliang Chen, Ruofei Yang, Zilong Hu, Shaozhong Wei, Sheng Hu, Xinjun Xiong, Meijuan Wang, Ammar Lubeiny, Xiaohua Li, Minglei Feng, Shuang Dong, Xinlu Xie, Chao Nie, Jingyi Zhang, Yunhao Luo, Yichen Zhou, Ruodi Liu, Jinhai Pan, De-Xin Kong, Xuebo Hu, A terpenoids database with the chemical content as a novel agronomic trait, Database, Volume 2024, 2024, baae027, https://doi.org/10.1093/database/baae027

In the originally published version of this article, the legends of Figure 2, Figure 4, Figure 5, Figure 6, Figure 7 and Figure 9 were incomplete.

The Publisher apologizes for this error, which has been corrected.

