# Supplementary figures and images for: A terpenoids database with the chemical content as a novel agronomic trait
Source: Database (Oxford). 2024 May 22;2024:baae027. doi: 10.1093/database/baae027 (PMC11110934; doi:10.1093/database/baae027)

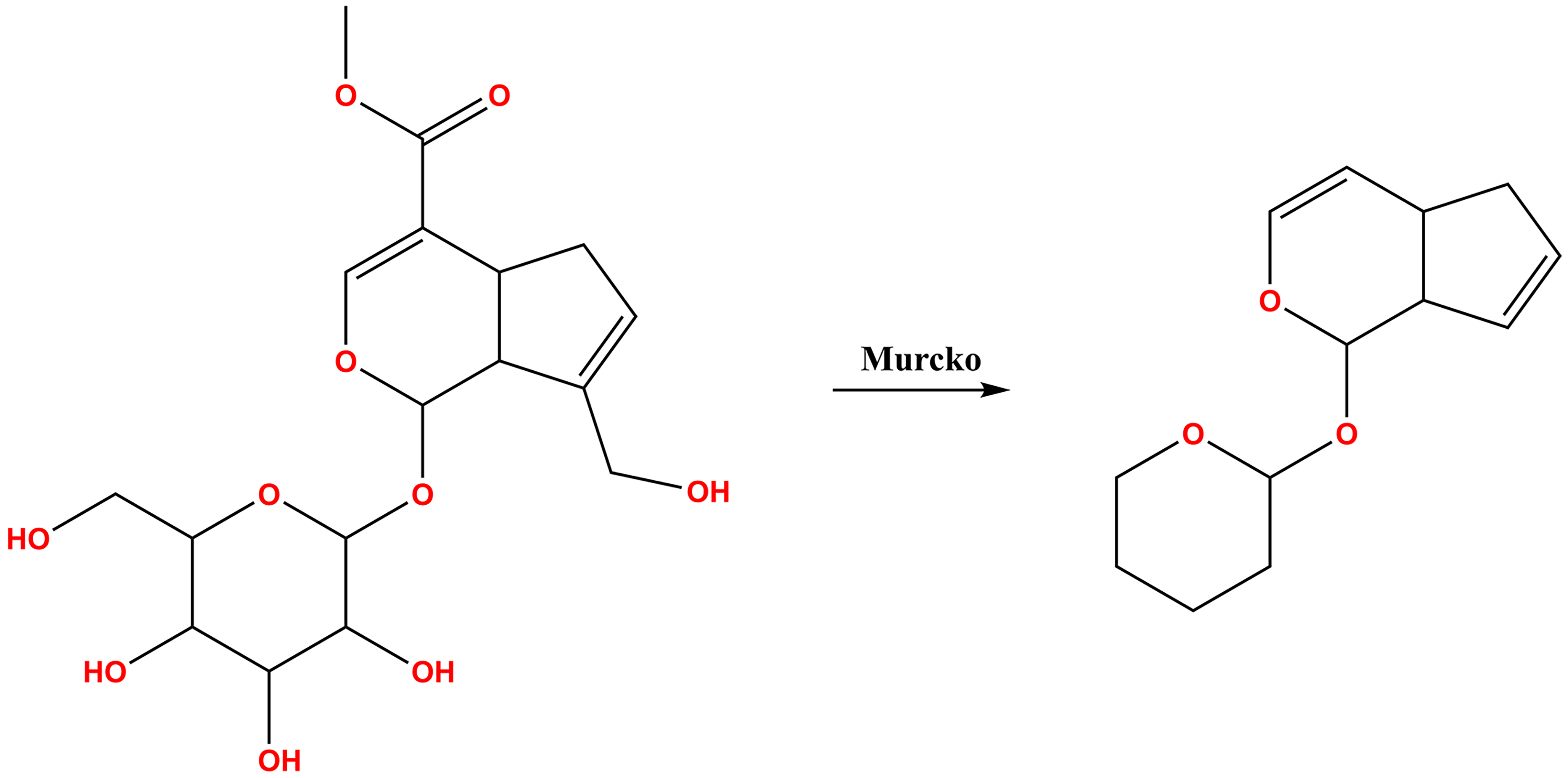

Supplement: baae027_Supp [file baae027_supp.zip › suppl_data/Supplementary Figure S1.tif]

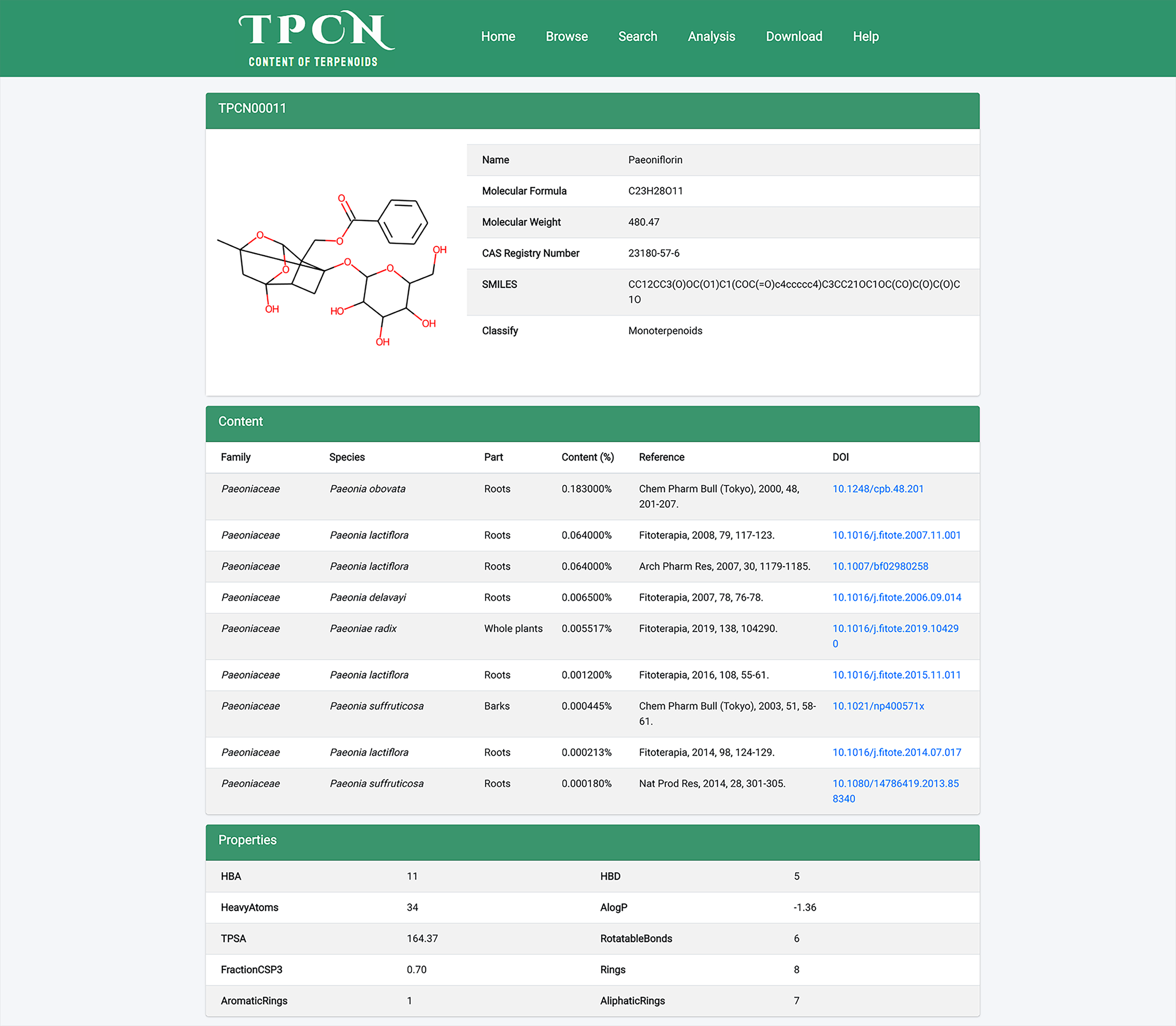

Supplement: baae027_Supp [file baae027_supp.zip › suppl_data/Supplementary Figure S2.tif]

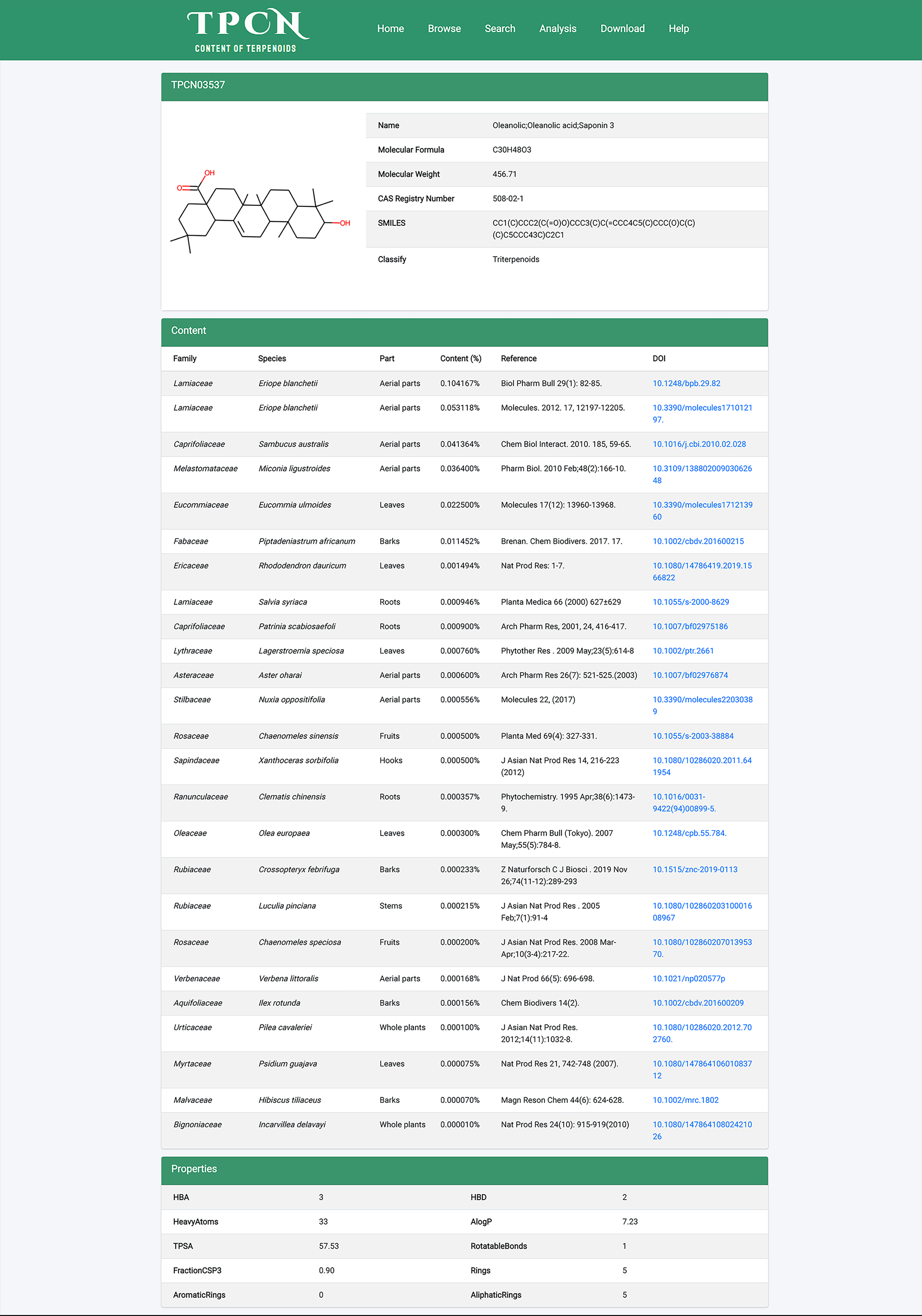

Supplement: baae027_Supp [file baae027_supp.zip › suppl_data/Supplementary Figure S3.tif]
